# Ankle fracture with syndesmotic injury treated by screw fixation: a prospective study on clinical and radiographic outcomes

**DOI:** 10.3389/fsurg.2025.1689228

**Published:** 2025-10-09

**Authors:** Xiaodong Li, Pengcheng Liu, Ran Duan, Xiang Wang, Qianhua Zhu, Shuangling Ni, Xiaoqing Wang

**Affiliations:** 1 Department of Orthopedics, Shanghai Key Laboratory of Orthopedic Implant, Shanghai Ninth People's Hospital, Shanghai Jiao Tong University School of Medicine, Shanghai, China; 2 Department of Plastic and Reconstructive Surgery, Shanghai Ninth People's Hospital, Shanghai Jiao Tong University School of Medicine, Shanghai, China; 3 Department of Nursing, Shanghai Ninth People's Hospital, Shanghai Jiao Tong University School of Medicine, Shanghai, China

**Keywords:** ankle fracture, syndesmotic fixation, computed tomography, syndesmosis, malreduction

## Abstract

**Purpose:**

The syndesmotic screws are frequently utilized in the treatment of unstable ankle fractures. However, significant controversies persist regarding their optimal application strategies. This study aims to investigate the dynamic changes in syndesmotic reduction among patients with unstable ankle fractures following syndesmotic screw fixation and to explore the relationship between malreduction and functional outcomes.

**Methods:**

Patients with unstable ankle fractures who underwent open reduction and internal fixation (ORIF) with syndesmotic screw fixation from January 2020 were prospectively enrolled. Syndesmotic screws were routinely removed 8–12 weeks post-fixation. All patients were followed up at five time points: immediately after internal fixation, prior to syndesmotic screw removal (8–12 weeks), and at 3, 6, and 12 months post-initial fixation. Evaluations included imaging (Computed Tomography, CT), functional outcomes [Ankle and Hindfoot Function Scoring System (AOFAS), Olerud-Molander Ankle Score (OMAS)], and pain assessment (Visual Analog Scale, VAS).

**Results:**

From January 2020 to January 2021, a total of 26 patients were included in this study. The incidence of malreduction at the five follow-up time points was 69.2% (18/26), 61.5% (17/26), 50% (13/26), 61.5% (16/26), and 61.5% (16/26), respectively. Malreduction of the anterior tibiofibular distance and fibular rotation were the primary contributing factors. Functional outcomes were significantly worse for patients with malreduction following syndesmotic screw removal compared to those without malreduction (*p* < 0.05).

**Conclusions:**

(1) Dynamic changes in syndesmotic reduction were observed at various time points within one year post-surgery. Removal of the syndesmotic screw improved syndesmotic reduction to some extent; however, re-diastasis may occur after weight-bearing. (2) Syndesmotic malreduction following screw removal was associated with poorer ankle functional outcomes.

## 1 Introduction

Ankle fractures are among the most prevalent fractures encountered in the emergency department, frequently accompanied by distal tibiofibular syndesmosis injury and ankle instability. According to reports, approximately 5%–10% of ankle sprain injuries and 10%–20% of ankle fractures involve distal tibiofibular syndesmosis injury, which typically requires surgical intervention (1–8). The current standard treatment involves using syndesmotic screws (SS) to stabilize the distal tibiofibular syndesmosis following open reduction and internal fixation (ORIF) for fractures (9–14). However, several aspects remain controversial, including the optimal diameter, position, and number of SS, whether and when the screw should be removed, and when weight-bearing should commence (15–21).

Malreduction of the distal tibiofibular syndesmosis is recognized as a common complication of unstable ankles, potentially leading to early ankle dysfunction and long-term traumatic arthritis (6, 22). Previous studies have explored the use of SS for reduction. Gardner et al. retrospectively evaluated 25 patients with ankle fractures and syndesmotic instability who underwent ORIF, finding that 52% exhibited evidence of syndesmotic malreduction on postoperative CT imaging. Endo et al. demonstrated that the anterior tibiofibular distance widened one year after syndesmotic screw removal (23). Nevertheless, these studies assessed syndesmotic reduction solely from an imaging perspective, without considering patient function. Consequently, they fail to provide clinically meaningful recommendations.

Currently, there remains a paucity of observations regarding continuous imaging changes in ankles following syndesmotic screw fixation, including reduction after SS placement, changes in the tibiofibular distance before and after screw removal, and short-term and long-term changes in the tibiofibular syndesmosis after weight-bearing. Additionally, further investigation into the relationship between these dynamic changes and functional outcomes is essential.

The objectives of this prospective study were as follows: (1) To observe time-dependent changes in images and functions of ankles within one year in patients undergoing SS fixation; (2) To evaluate the effect of SS removal on the tibiofibular syndesmosis; (3) To determine the rate of syndesmotic malreduction before and after SS removal; and (4) To investigate the relationship between imaging changes and functional outcomes of the ankles.

To our knowledge, this study provides the most comprehensive follow-up imaging data within one year for patients with ankle fractures involving tibiofibular syndesmosis injury who underwent ORIF. Most prior studies selected only one or two time points for follow-up (23, 24). In contrast, this study evaluated five key time points to capture the most complete dynamic trend of tibiofibular syndesmosis changes.

## 2 Materials and methods

### 2.1 Patients

This study was approved by the Ethics Committee of our hospital, and all participants provided written informed consent. Patients were prospectively recruited at our hospital between January 2020 and January 2021. This study was conducted in accordance with the Strengthening the Reporting of Observational Studies in Epidemiology (STROBE) guidelines.

Inclusion criteria were as follows: patients aged 18–75 years, suffering from ankle fractures with diastasis of the tibiofibular syndesmosis or Maisonneuve fractures, and receiving ORIF with syndesmotic screw fixation within two weeks.

Exclusion criteria included open fractures, history of ankle injuries or surgeries, history of ankle osteoarthritis (Kellgren-Lawrence grade ≥2 based on x-ray), fractures accompanied by vascular or nerve injury, pathological fractures, concomitant fractures of the ipsilateral lower limb, and mental disorders precluding cooperation.

### 2.2 Surgical plan

All patients underwent ORIF according to AO principles. Distal fibula fractures were fixed with anatomical plates, and medial malleolus fractures were fixed with two cannulated screws (3.5 mm in diameter). Posterior malleolus fractures involving more than 25% of the articular surface were fixed with one to two cannulated screws, while those involving less than 25% were managed at the surgeon's discretion during the operation.

Following bone structure fixation, the stability of the distal tibiofibular syndesmosis was further assessed intraoperatively. The hook test was performed under C-arm fluoroscopy, and the tibiofibular distance or medial ankle joint space was measured bilaterally. A difference exceeding 2 mm indicated tibiofibular syndesmosis instability (25). Syndesmosis reduction was achieved under direct visualization. During SS insertion, the ankle was maintained in a neutral position, and the reduction clamp was used to temporarily fix the distal tibiofibular joint before screw insertion. Then, one SS (3.5 mm full-threaded cortical screw) was inserted from the fibula to the tibia. The optimal location for syndesmotic screw placement is typically 2–4 cm above the tibial plafond, parallel to the joint line, and engaging three or four cortices. For Maisonneuve injuries, the distal tibiofibular syndesmosis was stabilized using two syndesmotic screws (SS), and the deltoid ligament complex of the medial ankle was repaired with anchors. All SS were designed to penetrate three layers of the bone cortex. Patients were instructed to gradually perform non-weight-bearing functional exercises of the ankles until the SS were removed 8–12 weeks post-internal fixation (26). Following screw removal, gradual weight-bearing commenced on the injured limb (Supplementary Table S1).

### 2.3 Postoperative follow-up

**Figure 1:**
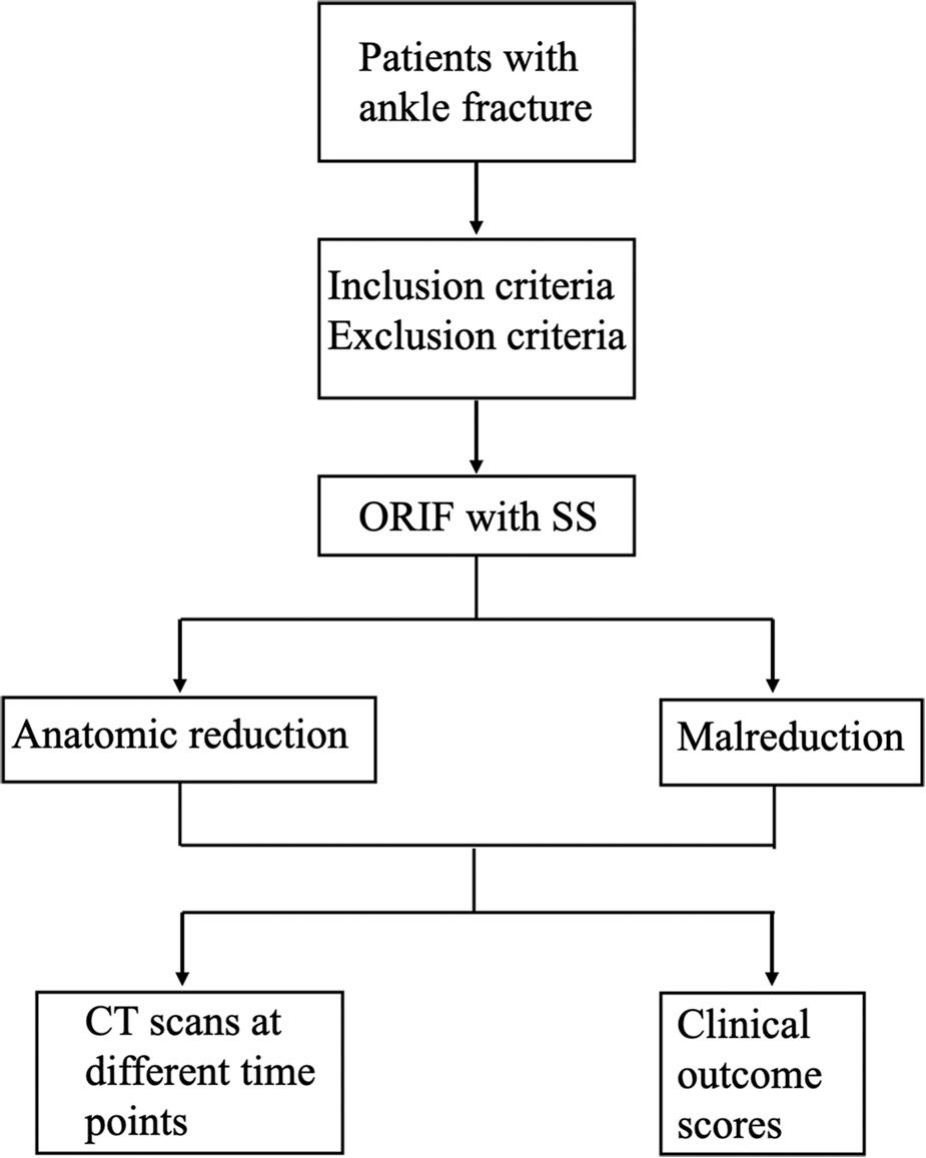
The research flow diagram.

All participants underwent follow-up assessments at five specific time points (Table 1): immediately after internal fixation, prior to syndesmotic screw removal, 3 months post-internal fixation, 6 months post-internal fixation, and 12 months post-internal fixation. Conventional ankle computed tomography (CT) scans were performed at these time points, including bilateral scans at each assessment. Using the uninjured side as a control, imaging parameters of the injured side were measured to evaluate the reduction of the tibiofibular syndesmosis. During each CT examination, non-scanned body parts were shielded with lead clothing to minimize radiation exposure. Additionally, the American Orthopedic Foot and Ankle Society Score (AOFAS), Olerud-Molander Ankle Score (OMAS), and Visual Analogue Scale (VAS) were utilized to assess functional outcomes at 3, 6, and 12 months post-internal fixation (Figure 1).

**Table 1:** The patient's postoperative follow-up status.

Timepoints	Enrollment	Follow up			
	Immediately after the internal fixation	8–12 weeks	3 months	6 months	12 months
Enrollment					
Screening	√				
Sign informed consent	√				
Interventions					
Removal of syndesmotic screws		√			
Assessment					
CT	√	√	√	√	√
		(before the removal of syndesmotic screws)	(after the removal of syndesmotic screws)		
AOFAS			√	√	√
OMAS			√	√	√
VAS			√	√	√

### 2.4 Imaging measurements of the distal tibiofibular syndesmosis

**Figure 2:**
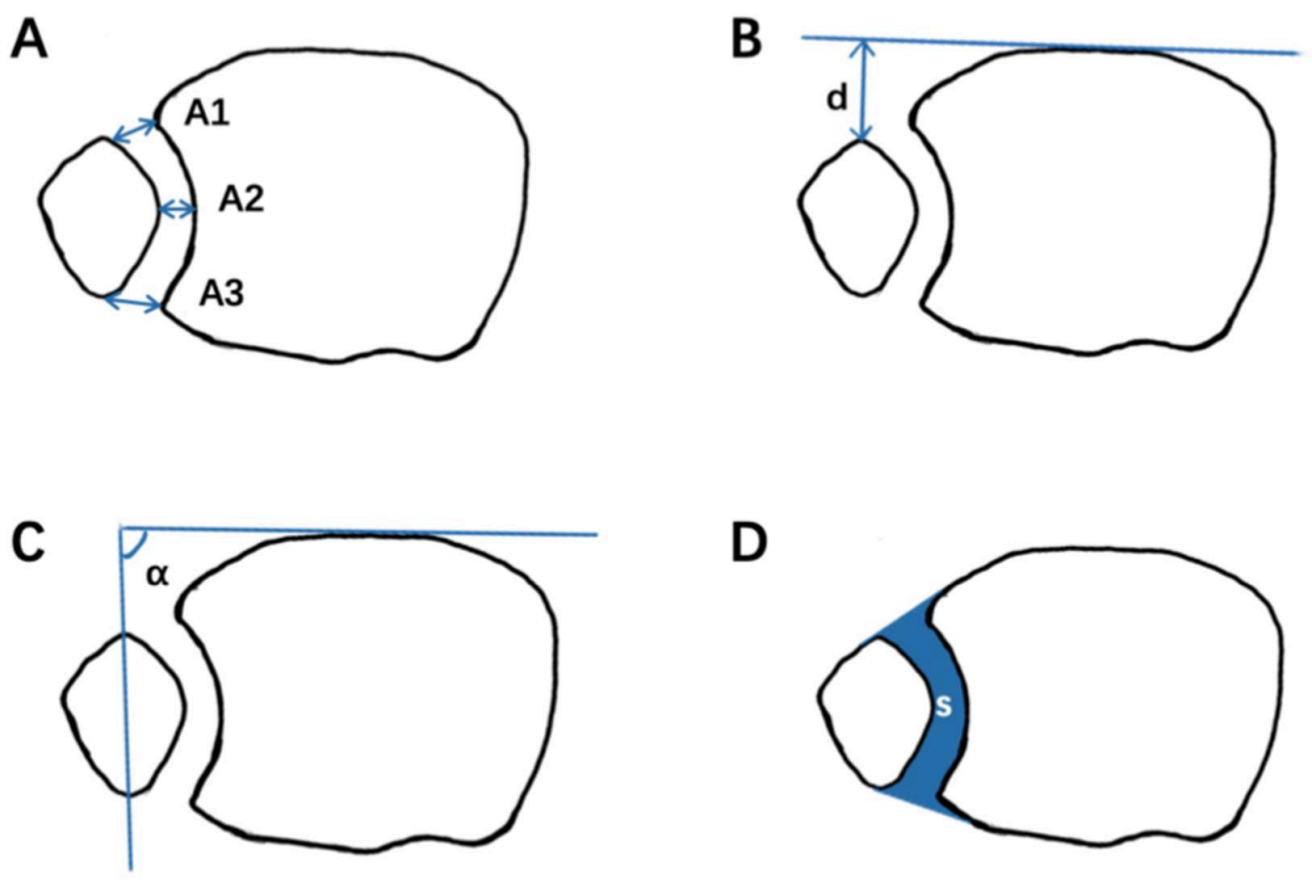
Measurements for evaluating syndesmotic reduction. **(A)** Illustration of an axial view of a normal syndesmosis proximal to the tibial plafond with measures A1 to A3. **(B)** Anteroposterior fibular translation. **(C)** Fibular rotation. **(D)** The sum of the syndesmosis area.

In this study, a 1 mm-thick CT scan was employed. Imaging data were stored in Digital Imaging and Communications in Medicine (DICOM) format and imported into RadiAnt Viewer software (V2021.2, Medixant, Promienista 2560-288 Poznań, Poland) for analysis. All measurements were conducted at a standard axial position 10 mm above the articular surface. The following parameters were assessed (Figure 2): anterior tibiofibular distance (ATD), central tibiofibular distance (CTD), posterior tibiofibular distance (PTD), fibular translation distance (FTD), and fibular rotation angle (FRA) (27–30). Furthermore, the syndesmosis area and syndesmosis volume were also quantified (31). The syndesmosis area was defined by the boundaries of the tibiofibular joint and its anterior and posterior edges. The syndesmosis volume was calculated layer-by-layer from the syndesmosis area extending 3 cm above the ankle joint, approximated as the sum of the syndesmosis area × thickness of layers: syndesmosis volume = [A1 × T] + [A2 × T] + [A3 × T] + … + [An × T], where A represents the syndesmosis area at each layer, and T represents the thickness of each layer (1 mm in this study).

Malreduction of the tibiofibular syndesmosis was defined as meeting any of the following criteria: (1) a side-to-side difference of 2 mm or more in at least one parameter, including ATD, CTD, PTD, FTD; (2) a side-to-side difference of 5 degrees or more in FRA (23).

Participants were categorized into two groups based on CT findings at different time points: malreduction and anatomic reduction. Functional outcomes at 12 months post-internal fixation were compared between the two groups. A senior orthopedic surgeon, who was not involved in the treatment, conducted all CT measurements. To confirm measurement repeatability, assessments were repeated at 12-week intervals. The results of the two measurements were evaluated using the Intraclass Correlation Coefficient (ICC). Comparisons were made between the malreduction and anatomical reduction groups at various time points. Additionally, a comparison was conducted between the affected and healthy limbs within each patient.

### 2.5 Statistical analysis

All data were analyzed using Tukey's multiple comparison *post-hoc* test and Wilcoxon signed-rank test via the Statistical Package for the Social Sciences (SPSS, version 26.0, IBM Corporation, Chicago, IL, USA). A *p*-value < 0.05 was considered statistically significant.

## 3 Results

Between January 2020 and January 2021, 179 patients with ankle fractures were hospitalized for open reduction and internal fixation (ORIF). Of these, 37 patients (20.6%) met the inclusion criteria; however, four patients declined informed consent, and seven patients dropped out during the one-year follow-up period. Ultimately, 26 eligible patients (17 males and 9 females, mean age 46 years, range 18–73 years) were included in the study (Table 2). Eleven patients had 44-B fractures, and 15 patients had 44-C fractures. According to the Lauge-Hansen classification, there were 11 cases of supination-external rotation fractures, 12 cases of pronation-external rotation fractures, and 3 cases of pronation-abduction fractures. Among these, 22 patients were stabilized with one syndesmotic screw, while the remaining four (Maisonneuve injuries) required two SS (Supplementary Table S2).

**Table 2:** Patients demographics and results.

Case	Sex	Age	Injury features	Operation	Complications
1	M	28	Supination-external rotation	ORIF	No
2	M	33	Supination-external rotation	ORIF	No
3	M	56	Supination-external rotation	ORIF	Pain
4	M	32	Supination-external rotation	ORIF	No
5	M	47	Supination-external rotation	ORIF	No
6	M	51	Pronation-external rotation	ORIF	No
7	F	45	Pronation-external rotation	ORIF	No
8	F	44	Pronation-external rotation	ORIF	No
9	M	68	Pronation-external rotation	ORIF	Pain
10	M	73	Supination-external rotation	ORIF	No
11	M	39	Pronation-abduction	ORIF	No
12	F	34	Supination-external rotation	ORIF	No
13	M	18	Pronation-external rotation	ORIF	No
14	F	30	Pronation-external rotation	ORIF	No
15	M	27	Pronation-external rotation	ORIF	No
16	M	63	Pronation-external rotation	ORIF	Pain
17	M	45	Supination-external rotation	ORIF	Pain
18	M	26	Pronation-external rotation	ORIF	No
19	F	47	Pronation-external rotation	ORIF	No
20	F	56	Supination-external rotation	ORIF	No
21	M	29	Supination-external rotation	ORIF	No
22	F	55	Supination-external rotation	ORIF	Pain
23	F	41	Pronation-abduction	ORIF	Pain
24	M	35	Pronation-abduction	ORIF	No
25	M	29	Pronation-external rotation	ORIF	No
26	F	37	Pronation-external rotation	ORIF	No

### 3.1 Imaging measurements

**Figure 3:**
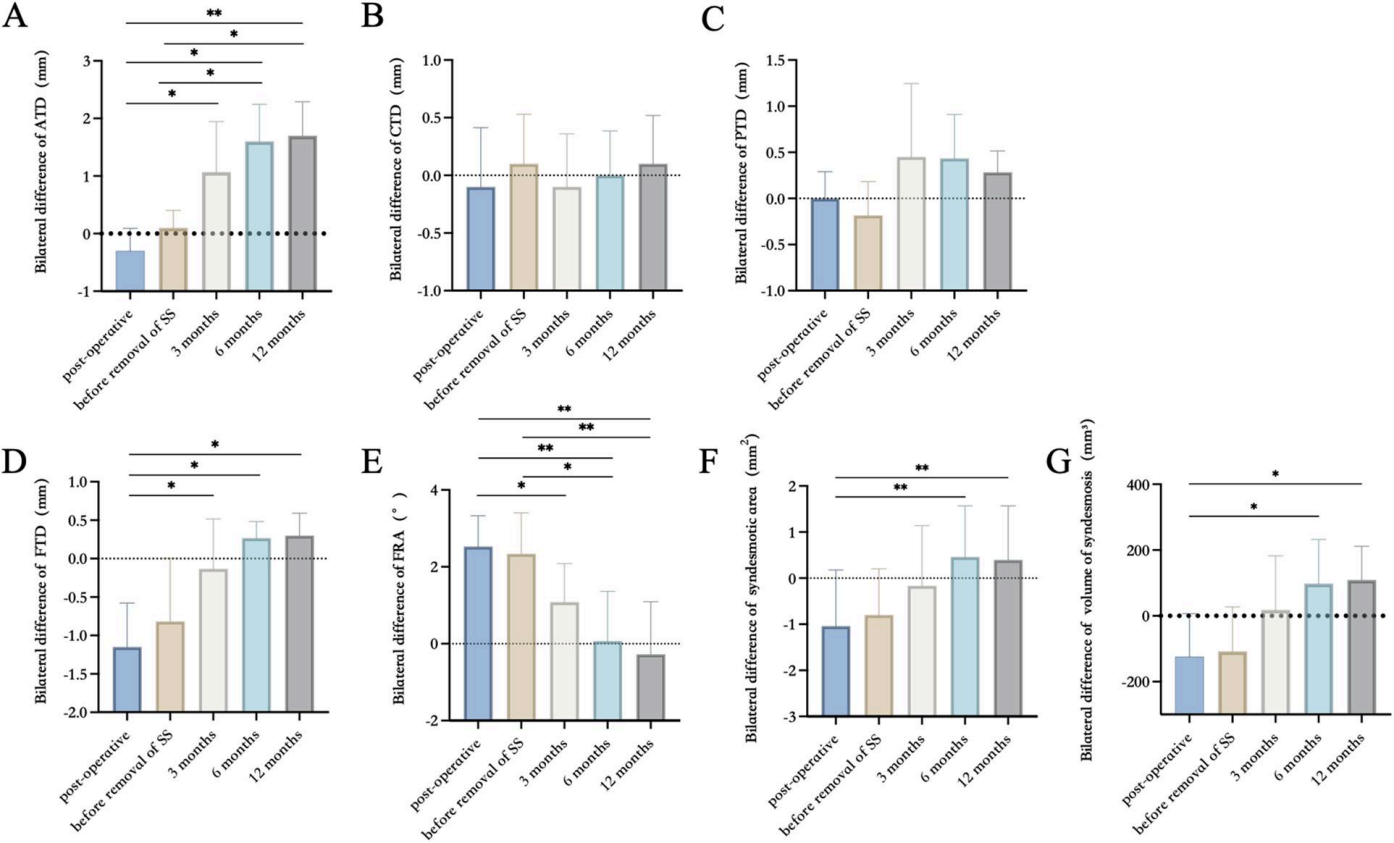
The results of imaging measurements in ATD, CTD, PTD, FTD, FRA, the sum of the syndesmosis area, and volume of syndesmosis. **(A)** Bilateral difference of ATD. **(B)** Bilateral difference of CTD. **(C)** Bilateral difference of PTD. **(D)** Bilateral difference of FTD. **(E)** Bilateral difference of FRA. **(F)** Bilateral difference of syndesmosis area. **(G)** Bilateral difference of volume of syndesmosis. Error bars present mean ± SD. *p < 0.05, **p < 0.01, and ***p < 0.001.

The results of imaging measurements are presented in Table 3 and Figure 3. Within one year post-internal fixation, the bilateral differences in ATD, FTD, FRA, syndesmosis area, and syndesmosis volume exhibited changes. No statistically significant differences were observed in the mean values of CTD and PTD across any time points within one year post-internal fixation. According to the definition of malreduction of the distal tibiofibular syndesmosis used in this study, the incidences of malreduction immediately after internal fixation, before removal of syndesmotic screws (SS), after removal of SS (3 months post-fixation), 6 months post-fixation, and 12 months post-fixation were 69.2% (18/26), 61.5% (17/26), 50% (13/26), 61.5% (16/26), and 61.5% (16/26), respectively. Among these, anterior tibiofibular distance malreduction and fibular rotational malreduction accounted for the majority.

**Table 3:** The results of imaging measurements.

Measurement item	Immediately after the internal fixation	The removal of syndesmotic screws	3 months	6 months	12 months
Anterior tibiofibular distance (mm)	−0.31 ± 1.53	0.14 ± 1.51	1.01 ± 1.34	1.67 ± 1.21	1.75 ± 1.16
Central tibiofibular distance (mm)	−0.13 ± 1.41	0.15 ± 1.23	−0.13 ± 1.09	0.03 ± 1.07	0.13 ± 1.26
Posterior tibiofibular distance (mm)	0.07 ± 1.12	−0.24 ± 1.34	0.53 ± 1.01	0.40 ± 0.82	0.36 ± 1.12
Anteroposterior fibular translation (mm)	−1.23 ± 1.51	−1.02 ± 1.56	−0.17 ± 1.22	0.31 ± 1.06	0.33 ± 1.14
Fibular rotation (°)	2.53 ± 2.66	2.32 ± 2.80	1.12 ± 3.68	0.01 ± 3.25	−0.25 ± 2.81
Syndesmosis area (mm^2^)	−1.14 ± 7.51	−0.82 ± 8.13	−0.26 ± 7.70	0.54 ± 7.57	0.46 ± 8.41
Syndesmosis volume (mm^3^)	−124.65 ± 98.23	−108.02 ± 105.14	18.34 ± 112.60	98.01 ± 86.12	108.47 ± 81.38

All the data were the average value of side-to-side difference. Values indicate mean ± standard deviation.

### 3.2 Functional follow-up

The median AOFAS scores at 3, 6, and 12 months following internal fixation were 53 (IQR: 28), 80 (IQR: 18), and 85 (IQR: 18), respectively. The median OMAS scores at 3, 6, and 12 month post-fixation were 37.5 (IQR: 15), 75 (IQR: 20), and 85 (IQR: 20), respectively. The average VAS scores at 3, 6, and 12 month post-fixation were 2.43, 1.63, and 1.54, respectively.

All patients were divided into two groups based on immediate postoperative CT scans of their ankles: anatomic reduction and malreduction. At the 12-month follow-up, the anatomic reduction group exhibited higher AOFAS, OMAS, and VAS scores compared to the malreduction group; however, no statistically significant differences were observed. Subsequently, according to ankle CT scans taken at 3 months and 12 months post-fixation, all patients were again categorized into anatomic reduction and malreduction groups. The results demonstrated a significant difference in functional and pain scores at 12 months post-fixation (Table 4).

**Table 4:** The results of functional follow-up based on postoperative CT scans.

Group	Clinical outcome scores	Malreduction group	Anatomic reduction group	*P* value
Immediate postoperative CT scans	AOFAS	85.2	87.5	.38
	OMAS	84.3	88	.36
	VAS	1.4	1.2	.89
CT scans at 3 months post-fixation	AOFAS	83.1	89.7	.05
	OMAS	82.5	90.0	.02
	VAS	1.7	1.4	<.01
CT scans at 12 months post-fixation	AOFAS	82.9	91.0	.01
	OMAS	81.6	93.0	<.01
	VAS	1.8	0.9	<.01

## 4 Discussion

This study revealed that the absolute bilateral differences in anterior tibiofibular distance (ATD), fibular rotation angle (FRA), syndesmosis area, and syndesmosis volume significantly decreased after the removal of syndesmotic screws. Additionally, the fibula shifted posteriorly and rotated externally, potentially due to overcompression of the tibiofibular syndesmosis during SS implantation in some patients. Automatic reduction of the ankle occurred after SS removal. With the initiation of weight-bearing exercises, the absolute side-to-side differences in these parameters gradually increased and stabilized by six months post-fixation.

Based on these measurements, we can more accurately assess the reduction quality of the tibiofibular syndesmosis in patients. In this study, the immediate postoperative rate of syndesmotic malreduction reached 69.2%, likely attributable to overcompression of the tibiofibular syndesmosis. Following SS removal, the malreduction rate decreased. Several studies have reported syndesmotic overcompression and external rotation after fixation of ankle fractures with syndesmotic injury when using reduction forceps, particularly noticeable in elderly patients with osteoporosis (32–35). Cosgrove et al. emphasized the importance of the position of the medial clamp tine during syndesmotic reduction using forceps (36). Regauer et al. suggested that the stability of the posterior malleolus, as well as the medial and lateral collateral ankle ligaments, are critical determinants of tibiofibular reduction quality and recommended avoiding the use of reduction clamps or forceps whenever possible (37). Furthermore, surgeons' tactile feedback during SS insertion into the bone cortex also plays a subjective role in assessing potential overcompression during tibiofibular syndesmosis reduction.

Anatomic reduction of the distal tibiofibular syndesmosis is crucial for long-term functional outcomes (38–40). Numerous clinical studies have investigated postoperative changes in the tibiofibular syndesmosis, with varying results depending on time points and treatment strategies. Song et al. evaluated postoperative CT scans of bilateral ankles within two weeks of internal fixation and 30 days after SS removal (41). All SS were removed three months post-fixation. Results indicated that nine patients (36%) exhibited evidence of tibiofibular syndesmosis malreduction on initial postoperative axial CT scans. After SS removal, 8 out of 9 (89%) cases showed adequate reduction of the tibiofibular syndesmosis based on CT findings. Conversely, Kortekangas et al. utilized intraoperative CT with O-arm and postoperative CT of bilateral ankles under weight-bearing conditions at two-year follow-up to assess tibiofibular syndesmosis reduction (42). SS were not removed in any patients. Results showed that the rate of syndesmotic malreduction with SS increased from 5% (1/21)–16% (3/19) after two years. Comparatively, as reported by Endo et al., all patients had their SS removed six weeks post-fixation, and the rate of syndesmotic malreduction increased from 50% immediately post-fixation to 60% one year later (23).

In this study, patients were categorized into two groups—malreduction and anatomical reduction—based on computed tomography (CT) scans obtained three months after initial fixation following the removal of syndesmotic screws (SS). Functional outcomes at 12 months post-internal fixation were compared between the two groups. The results revealed statistically significant differences in AOFAS, OMAS, and VAS scores (*p* = .05, *p* = .02, *p* < .01), suggesting that malreduction of the syndesmosis after SS removal may negatively affect functional recovery.

Although ankle CT scans have been widely accepted by surgeons as a reliable method for evaluating tibiofibular syndesmosis, they are typically performed only before and after internal fixation, with limited attention given to post-removal assessment. Sagi et al. reported that patients with syndesmotic malreduction exhibited significantly worse functional outcomes at a two-year follow-up based on postoperative CT findings (40). Consequently, they recommended comparing postoperative CT scans with those of the contralateral limb to improve diagnostic accuracy. In contrast, this study emphasizes the importance of conducting CT evaluations after SS removal. Due to the stabilizing effect of the SS, some patients may maintain an anatomical reduction immediately after surgery; however, malreduction or diastasis may become apparent only after screw removal. Therefore, we recommend routine CT scanning following SS removal to enable early detection of syndesmotic abnormalities and timely intervention.

The optimal timing and necessity of SS removal remain controversial. Some studies advocate removing the screws six to eight weeks postoperatively to facilitate ligament healing and promote early weight-bearing, which has been shown to enhance functional outcomes (23). Conversely, other researchers support a more conservative approach, advising non-weight-bearing activity until 12 weeks or longer after surgery to prevent late syndesmotic diastasis, particularly in cases involving Weber C fractures. Furthermore, systematic reviews have found no significant difference in functional outcomes between routine and on-demand SS removal (43–45). Thus, it is suggested that SS removal should be considered only when patients present symptoms such as pain, mobility impairment, or infection (9, 46–48).

This study has several limitations. Although it was designed as a prospective cohort study, the sample size was relatively small due to a short enrollment period, multiple follow-up time points, and the impact of the COVID-19 pandemic. Among the 37 eligible patients enrolled in January 2020, four declined to provide informed consent (10.8%, 4/37), and seven were lost to follow-up (18.9%, 7/37). Potential reasons include the high frequency of required follow-ups (five times within one year), which many patients found burdensome after regaining normal ambulatory function within three to six months post-surgery. Additionally, the ongoing pandemic restricted patient mobility and discouraged hospital visits due to concerns about infection risk.

Another limitation pertains to the relatively short observation period. As noted by Egol et al., maximal functional recovery after ankle fracture surgery is typically achieved within one year (49). Our imaging data also indicated that syndesmotic parameters stabilized around six months post-fixation.

Future research will aim to address these limitations by including a control group comprising patients who did not undergo SS fixation and by strictly standardizing the timing of SS removal to enhance intergroup homogeneity. Moreover, gait analysis and weight-bearing CT scans will be incorporated into follow-up protocols to allow for a more comprehensive evaluation of syndesmotic integrity and functional outcomes.

## 5 Conclusion

1. Tibiofibular syndesmosis exhibits time-dependent changes within one year following internal fixation and SS removal. While SS removal may reduce the incidence of syndesmotic malreduction, syndesmotic diastasis may recur upon initiation of weight-bearing activities.
2. Malreduction of the tibiofibular syndesmosis following internal fixation was primarily characterized by a reduced anterior tibiofibular distance, internal rotation of the fibula, and anterior displacement of the fibula. This phenomenon may be associated with excessive compression of the tibiofibular syndesmosis by screws or improper screw insertion angles.
3. Routine postoperative CT evaluation is recommended after removal of the syndesmotic screw (SS). Furthermore, corrective intervention should be considered if malreduction is detected following screw removal.

## Data Availability

The original contributions presented in the study are included in the article/Supplementary Material, further inquiries can be directed to the corresponding author.
