# Supplementary material for: Ankle fracture with syndesmotic injury treated by screw fixation: a prospective study on clinical and radiographic outcomes
Source: Front Surg. 2025 Oct 9;12:1689228. doi: 10.3389/fsurg.2025.1689228 (PMC12547989; doi:10.3389/fsurg.2025.1689228)
Supplement: Supplementary file 1 [file Table1.docx]

Based on preoperative imaging findings and intraoperative physical examination, it was confirmed that all enrolled patients required fixation of the inferior tibiofibular syndesmosis using a syndesmotic tension screw. Postoperative rehabilitation shall be conducted according to the following protocol (Supplementary Table 1). The **early postoperative phase is about 0-6weeks.** The objectives of this phase are to control swelling and pain, prevent muscle atrophy, and maintain function in other parts of the body. Non-weight-bearing functional exercises are emphasized. The most essential and safe early exercise is ankle pumps. Performed lying or sitting, the patient maximally dorsiflexes and plantarflexes the ankle. Each position is held for 5–10 seconds; 20–30 repetitions per set, multiple sets daily. This “pump-like” motion promotes circulation, reduces swelling, and helps prevent thrombosis. Additional exercises include toe movements, knee flexion and extension, and hip/core training such as straight leg raises. The **intermediate phase is about 6–12 weeks. The objectives of this phase are** gradually resuming weight-bearing under medical guidance; restore joint range of motion; improve muscle strength. With physician approval, partial weight-bearing begins using crutches (e.g., 25% ,50%, and 75% body weight), progressing to one crutch and eventually full weight-bearing. In addition, active/assisted exercises within a pain-free range, including continued ankle pumps and towel-assisted inversion/eversion Isometric exercises such as pushing the foot inward, outward, upward, or downward against a fixed object (e.g., a wall or the opposite hand), holding for 5–10 seconds per contraction. The late phase is about after 12 weeks. **The objectives of this phase are to** enhance muscle strength, endurance, and balance; prepare to return to normal walking, work, and physical activity. Heel raises, squats, and use of equipment such as leg press machines and stationary bicycles (initially without resistance). In addition, **balance and proprioception training is also very important.** Progress from double-leg to single-leg standing, eventually incorporating unstable surfaces (e.g., soft mats) for increased challenge.

Supplementary Table 1. The patient's postoperative rehabilitation progress

|  | **Weight-bearing** | **Physical Activity** | **Strength Training** |
| --- | --- | --- | --- |
| **Early postoperative phase (0-6 weeks)** | **Non-weight-bearing (NWB)** | **Ankle Pumps** | **Straight leg raise** |
| **Intermediate phase (6-12 weeks)** | **Partial weight-bearing (PWB)** | **PWB with crutches** | **Isometric exercise** |
| Late phase (≥12 weeks) | **Full weight-bearing (FWB)** | **Gait Training** | **Heel-Raised Squat** |
