# Supplementary material for: Ankle fracture with syndesmotic injury treated by screw fixation: a prospective study on clinical and radiographic outcomes
Source: Front Surg. 2025 Oct 9;12:1689228. doi: 10.3389/fsurg.2025.1689228 (PMC12547989; doi:10.3389/fsurg.2025.1689228)
Supplement: Supplementary file 2 [file Table2.docx]

Supplementary Table 2. Baseline characteristics of study participants by subgroup

| Characteristic | Overall | Malreduction | Anatomic reduction |
| --- | --- | --- | --- |
| Age, years (Mean)  Sex, n (%) Male  Female  Fracture type  Supination-external rotation  Pronation-external rotation  Pronation-abduction | 41.8  65.4  34.6  11  12  3 | 37.5  56.3  43.7  37.5  50.0  12.5 | 48.8  80.0  20.0  50.0  40.0  10.0 |
